# Job Resources and Core Self-Evaluation as Predictors of Nurse Engagement and Patient-Safety Outcomes: A Longitudinal Study

**DOI:** 10.1155/2024/6693274

**Published:** 2024-01-29

**Authors:** Dan Luo, Xuening Yang, Yamei Bai, Yulei Song, Baoyun Chen, Ya Liu

**Affiliations:** ^1^School of Nursing, Nanjing University of Chinese Medicine, Nanjing, Jiangsu, China; ^2^Nursing Department, Xuzhou Central Hospital, Xuzhou, Jiangsu, China

## Abstract

**Background:**

Work engagement and patient-safety outcomes in nursing practice are critically significant. However, most previous studies evaluating antecedents of work engagement and patient-safety outcomes have used cross-sectional designs.

**Aims:**

To investigate the effects of job resources (organizational support and leader empowerment) and core self-evaluation on nurses' work engagement and patient-safety outcomes.

**Methods:**

This longitudinal study surveyed 2,618 registered nurses from 17 public hospitals in XuZhou, China. Participants completed self-report questionnaires on organizational support, leader empowerment, and core self-evaluation at baseline. Work engagement and patient-safety outcomes were collected 18 months after the baseline. The mixed linear regression and Johnson–Neyman statistical analysis were used to analyze data.

**Results:**

Organizational support was an outsize predictor of nurses' work engagement, followed by core self-evaluation and leader empowerment. Organizational support and core self-evaluation were equally crucial for predicting patient-safety outcomes. Moreover, the positive impact of leader empowerment on patient-safety outcomes became significant when the core self-evaluation score was below 51.

**Conclusions:**

This study demonstrated that organizational support, leader empowerment, and core self-evaluation are important determinants of nurses' work engagement and patient-safety outcomes. *Implications for Nursing Management*. Hospital managers and nurse leaders should consider providing multiple supports to motivate staff nurses to engage in work. When nurses' core self-evaluation is low, empowering training for nurse leaders should be essential to reduce adverse patient events.

## 1. Background

Work engagement has stimulated constant interest as it is closely related to nurses' work effectiveness, patient outcomes, and institutional costs [1]. Work engagement is a positive, fulfilling, work-related state of mind with three fundamental dimensions: vigor, dedication, and absorption [2]. International Council of Nurses has recognized the need for nurses committed to high-quality standards and engaged in their work in the implementation of global action on patient safety and achieving universal health coverage [3]. However, the level of engagement among nurses is often reported to be low, and the COVID-19 pandemic has worsened it [4].

The literature shows that both job and individual-level resources are essential predictors of nurses' work engagement [5]. Organizational support is a kind of job resource, which provides resources, reinforcement, encouragement, and communication to employees [6]. Nurses who experience more organizational support report higher work performance and engagement levels [7, 8]. In addition, empowerment is also a precious job resource that plays a crucial role in the professional growth of nurses [9]. Leader empowerment is defined as the giving or delegation of power and authority. Several studies have shown that nurse leaders' empowering behaviors, specifically psychological and structural empowerment, positively affect nurses' work engagement [10, 11].

Personality traits such as core self-evaluation are also closely correlated with work engagement among nurses. Core self-evaluation describes an individual's evaluation of themselves and combines four core traits: self-esteem, generalized self-efficacy, neuroticism, and locus of control [12]. A significant and expanding body of research in business and psychology correlates higher levels of employees' core self-evaluation with better work engagement [13]. Previous research on nurses' personality traits and work engagement has focused on optimism, resilience, introspection, sensibility, and hardiness [14, 15]. Though limited studies have examined the effect of core self-evaluation on work engagement among nurses, studies have shown that core self-evaluation is significantly associated with burnout [16], the polar opposite of engagement. According to the Nursing Job Demands-Resource (JD-R) Model [17], adequate job resources, combined with more incredible personal strengths, increase the possibility of nurses experiencing higher levels of work engagement. However, no empirical study has explored the interaction effects of organizational support or leader empowerment and core self-evaluation on the engagement of nurses. Based on these theoretical and empirical arguments, we hypothesize the following points:

 
*Hypothesis 1a*. Organizational support will predict nurses' work engagement
 
*Hypothesis 1b*. Leader empowerment will predict nurses' work engagement
 
*Hypothesis 1c*. Core self-evaluation will predict nurses' work engagement
 
*Hypothesis 1d*. The interaction effect of organizational support and core self-evaluation on nurses' work engagement will be statistically significant
 
*Hypothesis 1e*. The interaction effect of leader empowerment and core self-evaluation on nurses' work engagement will be statistically significant

An emergent amount of evidence supports that nurses' work engagement is associated with patient-safety outcomes [18, 19]. Typical adverse patient-safety outcomes formulated by the American Nurses Association include falls, pressure ulcers, healthcare-associated infections, medication errors, patient complaints, and verbal abuse [20]. One recent scoping review estimated that a median of 10% of patients were affected by at least one adverse event [21], which led to death and high medical costs. Organizational support is known as an important resource to enhance nurses' job performance [22], which plays a central role in promoting patient safety. However, few empirical studies have directly explored the relationship between organizational support and patient-safety outcomes. Researchers have linked patient-safety outcomes to leader empowerment. A review of the literature indicates that workplace empowerment is critical for nurses to implement person-centered care and promote patient safety [23]. Correctly identifying the patient before conducting any nursing operations can be fundamental to ensuring patient safety in hospitals. Kim and Kim found that leader empowerment was positively associated with nurses' patient identification behaviors [24].

Core self-evaluation has proven to be an essential predictor of nurses' clinical decision-making [25], which can guarantee patient safety [26]. However, no studies have directly explored the relationship between nurses' core self-evaluation and patient-safety outcomes. Furthermore, there exists a significant knowledge gap about how job resources in nursing affect patient-safety outcomes from the perspective of core self-evaluation. The following hypotheses are derived from these empirical arguments:

 
*Hypothesis 2a.* Organizational support will predict patient-safety outcomes
 
*Hypothesis 2b*. Leader empowerment will predict patient-safety outcomes
 
*Hypothesis 2c*. Core self-evaluation will predict patient-safety outcomes
 
*Hypothesis 2d*. The interaction effect of organizational support and core self-evaluation on patient-safety outcomes will be statistically significant
 
*Hypothesis 2e*. The interaction effect of leader empowerment and core self-evaluation on patient-safety outcomes will be statistically significant

**Figure 1:**
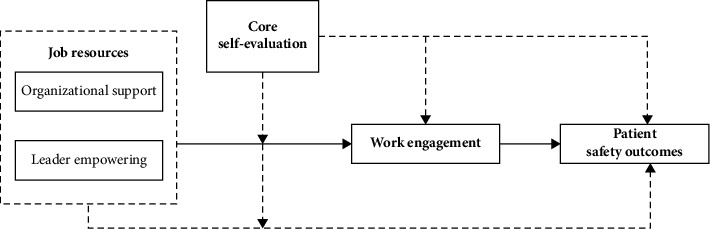
The theoretical framework of the relationship between job resources, core-evaluation, engagement, and patient-safety outcomes. A solid line indicates that the findings from the previous studies support the demonstrated relationships. A broken line indicates that no research evidence in nursing studies has demonstrated the relationships yet.

In summary, research on nurses' work engagement and patient-safety outcomes has increased over the last decade. However, most existing studies used a cross-sectional design. Furthermore, significant variances in nurses' work engagement and patient-safety outcomes may occur between hospitals [27]. Previous studies did not consider this phenomenon [17], which may affect the conclusions' accuracy and reliability. Based on the “Nursing Job Demands-Resource Model” and empirical evidence, the conceptual framework of this study (Figure 1) was developed.

## 2. Methods

### 2.1. Design

An 18-month prospective design was used in this study. Demographic characteristics, organizational support, leader empowerment, and core self-evaluation were collected at baseline, and work engagement and patient-safety outcomes were collected 18 months after the baseline. Data were collected from September 2021 to March 2023.

### 2.2. Participants

Random cluster sampling was performed to recruit nurses from 17 public hospitals in Xuzhou, China. Fifteen wards were randomly selected from each of 17 hospitals. All registered nurses of the sampled wards were invited to participate. The inclusion criteria were (i) being employed full-time; (ii) having more than one year of nursing work experience; and (iii) currently working in direct patient care roles. The PASS 2008 statistical software (Utah, USA) was used to calculate the sample size. A sample size of 1,067 from a population of 11,154 nurses in 17 hospitals was required based on a confidence level of 95% and a confidence interval of 3. To ensure that at least 10% of nurses in each of the hospitals participated, the target sample size was adjusted to 1,116. The final sample size of this study was 2,628, which met the minimum sample size requirement.

### 2.3. Data Collection

The online survey form was used to reduce response biases such as social desirability [28]. At time 1 (10 September 2021), the invitation to participate, with a link to the web-based questionnaire, was sent to eligible nurses via e-mail by the researcher. Reminder letters were sent to nonresponders 12 hours later. The same procedure was followed at time 2 (13 March 2023) by the same researcher. Only nurses who responded at time 1 were sent a time 2 survey package. At time 1, surveys were sent to 10,338 nurses and 6,234 were returned (60.3% response rate). At time 2, 2,618 nurses who responded at time 1 completed surveys (40.2% response rate).

### 2.4. Data Collection Tools

Web-based self-reported questionnaires were used to collect demographic characteristics, organizational support, leader empowerment, core self-evaluation, and patient-safety outcomes.

#### 2.4.1. Organizational Support

The organizational support was measured using the Survey of Perceived Organizational Support (SPOS) [6], a 9-item single-dimension questionnaire. Participants rated items on a 7-point Likert scale ranging from 1 = “strongly disagree” to 7 = “strongly agree.” Items were added to create total scores, with increasing scores indicating higher perceptions of organizational support. SPOS is a valid and reliable instrument for Chinese nurses [29]. Cronbach's *α* in this sample was 0.92.

#### 2.4.2. Leader Empowerment

We used the Leadership Empowerment Behavior (LEB) scale to assess levels of leader empowerment [30]. The 12-item LEB scale consists of four subscales (enhancing the meaningfulness of work, fostering participation in decision-making, expressing confidence in high performance, and providing autonomy from bureaucratic constraints). Each subscale consists of three items rated on a 5-point scale ranging from 1 = “strongly disagree” to 5 = “strongly agree,” added to create total scores. Higher overall scores suggest more leader empowerment behaviors. The LEB scale has been used by nurses with good psychometric properties [31]. Cronbach's *α* in our study was 0.97.

#### 2.4.3. Core Self-Evaluation

The Core Self-Evaluations Scale (CSES) was used to measure the core self-evaluations of nurses [32]. It consists of 12 items rated on a 5-point Likert scale (1 = “strongly disagree” to 5 = “strongly agree”). Noteworthily, six items in the CSES scale were rated in reverse order. The total score is measured by summing all items, with higher scores suggesting a better core self-evaluation. Zhang et al. have demonstrated the reliability of CSES in Chinese nurses [33]. In our study, Cronbach's *α* was 0.88.

#### 2.4.4. Work Engagement

We measured nurses' work engagement using the Utrecht Work Engagement Scale (UWES) [2], a 17-item questionnaire comprising three subscales: vigor (six items), dedication (four items), and absorption (five items). Each item is rated on a 7-point scale (0 = “never” to 6 = “always”), with higher scores representing greater engagement. This tool has been validated and applied to Chinese nurses [34]. Cronbach's *α* in this sample was 0.97.

#### 2.4.5. Patient-Safety Outcomes

Patient-safety outcomes were measured by the Adverse Patient Events Scale (APES) [35], derived from the Nursing Quality Indicators formulated by the American Nurses Association. The APES contains six patient adverse event types: falls, pressure ulcers, healthcare-associated infections, medication errors, patient complaints, and verbal abuse. Nurses were asked to recall their experience of patient adverse events as a result of direct patient care provided by themselves over the past year. Response options ranged from 1 (never) to 7 (daily). Nurses' assessments of patient adverse events have been utilized extensively in nursing and health care [36–38], and the APES was found to have excellent psychometric properties with Cronbach's *α* of 0.93 [38]. In our study, Cronbach's *α* was 0.84.

#### 2.4.6. Demographic Variables

Sociodemographic and professional information included the age (years), gender (female, male), the highest education level (college degree or below, university degree, or above), marital status (single, married), duration of working in nursing (years), professional title (nurse, nurse practitioner, or above), and annual salary ($).

### 2.5. Ethical Considerations

Institutional Review Board approval (no. KZXY-LK-20210903-026) was obtained from the researcher's hospital before commencing the study.

### 2.6. Strategy to Control the COVID-19 Pandemic Effects

Although the study was conducted post-COVID-19 pandemic, this crisis contingency may affect the study findings. We have taken the following measures to ensure the reliability of the results. First, all study hospitals have received unified training on the medical treatment of COVID-19 and specialized training on critical care, ensuring consistency in crisis management capabilities. Second, during the study period, there were no clusters of COVID-19 cases in the area where the study hospitals were located. Third, all study hospitals had a special ward for COVID-19 patients, and nurses from this ward were not included in this study. Fourth, we used a mixed linear model to accommodate for within-hospital correlations. In addition, the hospital hierarchy, which reflects the comprehensive strength and crisis management ability of each research hospital, was controlled in the data analysis.

### 2.7. Data Analysis

The first author, uninvolved in data collection and thereby unable to identify participants, conducted the entire analysis to avoid researcher bias. Data were presented as means ± standard divisions (SDs) or *n* (%). Before the analysis, the histogram plot was used to determine whether the numeric variables showed a normal distribution. Next, descriptive statistics and *t*-tests were applied to show the distributions of demographic characteristics and the association with nurses' work engagement and patient-safety outcomes.

Because of the multilevel nested structure of the data (2618 nurses from nine different hospitals), we used a data-analysis method of mixed linear models. The dependent variables were nurses' work engagement and patient-safety outcomes at the 18-month follow-up. In Step 1, the control variables on the individual level (demographic variables that showed statistically significant in *t*-tests) and on the hospital level (hospital hierarchy) were entered into the regression model; in Step 2, explanatory variables (organizational support, leader empowerment, and core self-evaluation) were added; and in Step 3, the interaction effect of the explanatory variables was added. In the analysis, the hospitals were treated as random effects, and the other independent variables as fixed effects. The model fit was evaluated by degree of freedom (DF), log-likelihood (LL), and the Akaike information criterion (AIC) [39].

To explicate significant interactions, we used the Johnson–Neyman technique [40] to determine how the effect of independent variables on dependent variables varies from being significant or not based on the moderator's value. All analyses were performed using IBM SPSS (version 22.0; SPSS Inc.), with *P* < 0.05 being considered statistically significant.

## 3. Results

### 3.1. Participant Characteristics

Participants included 2,618 registered nurses recruited from 17 public hospitals. Nurses were 31.53 ± 6.98 years old with 9.69 years of nursing experience. Most were female (98.8%), and about 79% held a university degree or above. Nearly half (44.5%) of respondents had a nurse practitioner or above professional title, and only 38.3% had an annual salary of more than $8,430.

There are statistical differences in work engagement in age, education level, marital status, work years, professional title, and annual salary (Table 1). Moreover, nurses' age, marital status, work years, and annual salary were also associated with patient-safety outcomes (all *P* < 0.05).

### 3.2. Longitudinal Analyses for Nurses' Work Engagement

Table 2 shows the results of longitudinal analyses for nurses' work engagement. The random effect of the variance of the hospital level was significant, indicating that nurses' work engagement varied among hospitals. In Step 1, nurses with a college degree or below (*P*=0.001) or married (*P*=0.007) exhibited higher levels of work engagement at the 18-month follow-up.

#### 3.2.1. Job Resources

In Step 2, Hypothesis 1a and Hypothesis 1b were supported: greater organizational support (*β* = 0.57, *P* < 0.001) and leader empowerment (*β* = 0.23, *P* < 0.001) predicated increased work engagement when accounting for participant's age, education level, marital status, work years, professional title, salary, and hospital hierarchy. A unit improvement in organizational support was associated with an increase in work engagement score of 0.57, compared to a 0.23 increase associated with a unit improvement in leader empowerment.

#### 3.2.2. Core Self-Evaluation

Baseline core self-evaluation (*β* = 0.51, *P* < 0.001) predicated 18-month work engagement when accounting for individual- and hospital-level variables (Step 2), supporting Hypothesis 1c. One unit increase in a participant's core self-evaluation score was associated with an increase in work engagement score of 0.51. Taking into account standardized effect estimates, organizational support was an outsize predictor (0.31) of nurses' work engagement, followed by core self-evaluation (0.19) and leader empowerment (0.09).

#### 3.2.3. Joint Effects

The joint effects of core self-evaluation with organizational support (*P*=0.650) and leader empowerment (*P*=0.310) were insignificant; thus, the Hypothesis 1d and Hypothesis 1e were not supported. When two interaction terms were added, the model fit became worse, with the restricted log-likelihood increasing by 14.44. Therefore, no interactions were explored in the work engagement model.

### 3.3. Longitudinal Analyses for Patient-Safety Outcomes

Table 3 shows the results of longitudinal analyses for patient-safety outcomes. In Step 1, nurses who were married (*P*=0.005) or made $60,000 a year (*P*=0.008) reported fewer patient adverse events at 18-month follow-up.

#### 3.3.1. Job Resources

In Step 2, higher baseline organizational support predicated fewer 18-month adverse patient events when nurses' marital status, salary, and hospital hierarchy were adjusted for. When the interaction effect of leader empowerment and core self-evaluation was added (Step 3), organizational support was also significantly associated with patient-safety outcomes (*β* = −0.02, *P* < 0.001), supporting Hypothesis 2a. Moreover, the effect of leader empowerment on patient-safety outcomes was statistically significant (*β* = −0.07, *P*=0.012) when the interaction between leader empowerment and core self-evaluation was adjusted for (Step 3), supporting Hypothesis 2b. One unit increase in leader empowerment score was associated with a decrease in patient adverse events score of 0.07, 3.5 times that due to a unit improvement in organizational support.

#### 3.3.2. Core Self-Evaluation

Hypothesis 2c was supported: baseline core self-evaluation predicted 18-month patient-safety outcomes (Step 2), and this effect remained statistically significant (*β* = −0.10, *P*=0.001) after controlling for the interaction effect of leader empowerment and core self-evaluation (Step 3). One unit increase in a participant's core self-evaluation score was associated with a decrease in patient adverse events score of 0.10. Taking into account standardized effect estimates, organizational support (−0.12) and core self-evaluation (−0.12) were equally crucial for patient-safety outcomes, followed by leader empowerment (−0.03).

#### 3.3.3. Joint Effects

**Figure 2:**
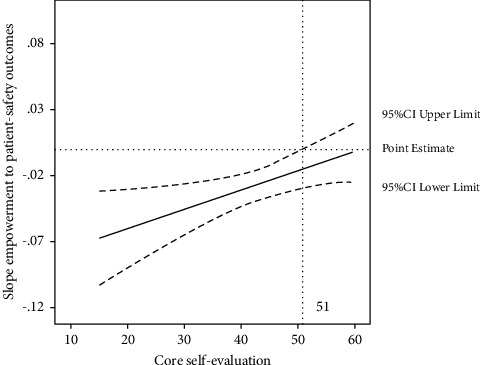
Estimated effect of leader empowerment on patient-safety outcomes moderated by core self-evaluation with Johnson–Neyman confidence bands.

After Step 2, the interaction terms of core self-evaluation with organizational support and leader empowerment were entered. Although the former was not significantly related to patient-safety outcomes, the latter was; thus, the Hypothesis 2d was not supported but the Hypothesis 2e was supported. Compared with only entering the interaction term of leader empowerment and core self-evaluation, entering the interaction terms of core self-evaluation with organizational support and leader empowerment made the model fit worse, with the restricted log-likelihood increasing by 12.89. Therefore, only the interaction between leader empowerment and core self-evaluation was explored in the patient-safety outcomes model (Step 3). The joint effect of leader empowerment and core self-evaluation on patient-safety outcomes was significant (*β* = 0.001, *P*=0.029). The results of the Johnson–Neyman analysis showed that the positive impact of leader empowerment on patient-safety outcomes became significant when the core self-evaluation score was <51 (Figure 2).

## 4. Discussion

In this study, we clarified the effects of organizational support, leader empowerment, and core self-evaluation on nurses' work engagement and adverse patient-safety outcomes among 2,618 nurses from 17 hospitals across 18 months and examined the interaction effects. Results showed that organizational support was an outsize predictor of nurses' work engagement, followed by core self-evaluation and leader empowerment. Organizational support and core self-evaluation were equally crucial for predicting patient-safety outcomes. Furthermore, the positive impact of leader empowerment on patient-safety outcomes became significant when the core self-evaluation score was below 51. These findings should enhance our understanding of the aspects of job resources and personality traits that influence nurses' work engagement and patient-safety outcomes at the hospital level.

Although research about nurses' work engagement has expanded over the decade, most published studies used cross-sectional study design. Several literature reviews identified methodological weaknesses and suggested that further research is required to decipher the antecedents of work engagement in nursing practice [17]. Our results are similar to previous findings on the positive effects of organizational support on engagement [7]. The material resources, fair rewards, and emotional encouragement from the hospital can boost the intrinsic interest of nurses in their work tasks [41]. There have been conflicting data on the impact of leader empowerment on engagement. In most quantitative studies, leader empowerment is positively associated with work engagement [10, 11]; however, some nurses reported negative perceptions of leader empowerment because communication around change initiatives was unclear and lacked feedback [42]. Our results supported that a higher level of leader empowerment leads to better work engagement in nurses. Therefore, effective communication and timely feedback for clinical nurses are essential for maintaining an empowering environment to ensure the engaged staff. Moreover, this study indicates that core self-evaluation is an independent predictor of nurses' work engagement. Several studies have demonstrated that a strong sense of self-efficacy (often described as a component of core self-evaluation) can help nurses continue to engage in clinical practice when they experience job stress and problems [7, 43]. Important information about the role of core self-evaluation in explaining work engagement among nurses needs to be included, as earlier studies almost focused on job satisfaction [44]. Work engagement has a stronger predictive value than job satisfaction since the former is closely related to nurses' care quality, patient outcomes, and institutional costs [1]. Regarding the contribution to the Nursing JD-R model [17], the present study added a new predictor-core self-evaluation. We also confirmed that leader empowerment is less strongly a predictor of work engagement than organizational support and core self-evaluation.

This study extended the finding of job resources and personality traits to outcomes other than nurses' work engagement, as we also focus on patient-safety outcomes. We found that organizational support positively affected patient-safety outcomes, which is consistent with results in existing studies [45]. Our finding suggested that adequate organizational support could improve work engagement in nurses and eventually decrease adverse patient-safety outcomes. Previous studies proved that nurses are more likely to be engaged in their work and provide high-quality care when the institutional structure and system support the care process [7, 46]. Another significant finding of our study pertains to the interaction effect of leader empowerment and core self-evaluation on patient-safety outcomes. More specifically, the benefits of high leader empowerment for fewer adverse patient events were only apparent in the context of low nurses' core self-evaluation. The possible explanation relates to the effect of leader empowerment on bridging the estrangement between leaders and employees caused by administrative hierarchy. A review noted that professional hierarchies in healthcare could increase the chance of communication failures and potentially harm patient safety [47]. According to the core self-evaluation theory [48], core self-evaluation affects employees' thinking processes and specific appraisals of job resources. Our results indicated that nurses with lower levels of core self-evaluation had a more substantial need for leader empowerment.

### 4.1. Limitations

Although our study applied longitudinal design and controlled confounding variables in the mixed linear regression model, it has several limitations. First, this study should be cautiously generalized since the participants were recruited from one geographic region. The use of a convenient sample of 13 hospitals and a response rate of 60% at baseline and 40% at the 2nd time point might suggest self-selection bias, thus threatening the external validity of this study. Additional studies should be conducted in other healthcare systems with more diverse participants. Another limitation is that nurses reported patient-safety outcomes. Although nurses' assessments of patient adverse events have been utilized extensively in nursing and health care and the measures have been proven to be valid and reliable, the reliability of the results might be compromised. Future studies should collect patient adverse event data from accurate records. Third, in order to gather longitudinal data, we did not utilize anonymous questionnaires, which might lead to social desirability responses. Despite we employed different researchers for data collection and analysis, the data remained susceptible to self-reporting bias. Lastly, while multiple strategies were used to reduce the impact of the COVID-19 pandemic on the study findings, we did not directly measure the crisis management ability of study hospitals, which may undermine the credibility of the results.

## 5. Relevance to Clinical Practice

The results of this study indicate that nurses' work engagement is mainly predicted by organizational support. Organizational-level interventions, encompassing theory- and evidence-based practices, should be available to nurses. Given that core self-evaluation is a personality trait that predicts both work engagement and patient-safety outcomes, it may be valuable to assess the baseline attributes of the candidates and commit to ongoing training to develop positive core self-evaluation attributes. Moreover, periodic assessments of nurses' work engagement, from the perspective of staff nurses, nurse leaders, physicians, and patients, may identify solid areas and those needing improvement relative to patient-safety outcomes. Lastly, the effect of leader empowerment should be emphasized, especially when staff nurse has a low level of core self-evaluation. Helping nurse leaders develop positive empowerment practices can be a potential strategy to enhance nurses' work engagement and prevent adverse patient events. Existing literature demonstrated practical empowerment skills for nurse leaders, including giving public praise, modeling behaviors, communication skills, and coaching abilities [49].

## 6. Conclusion

Organizational support, leader empowerment, and core self-evaluation significantly influenced nurses' work engagement and patient-safety outcomes. Although knowledge about the relevance of nurses' work engagement is accumulating, the present study confirmed the causality of relationships between the variables by longitudinal follow-up. We hope this study can provide evidence-based guidance for hospital managers to improve the engagement of nurses. Moreover, given the interaction effect of core self-evaluation and leader empowerment on patient-safety outcomes, empowerment training for nurse leaders should be an essential component of further interventions aiming to improve patient-safety outcomes when nurses have low core self-evaluation.

## Figures and Tables

**Table 1:** Baseline demographic characteristic differences in response to 18-month engagement and patient-safety outcomes (*N* = 2,618).

Characteristics	*N* (%)	Engagement	Safety outcomes
*Gender*			
Female	2586 (98.8)	82.42 ± 22.02	8.05 ± 2.46
Male	31 (1.2)	78.55 ± 21.56	8.19 ± 2.70
*P* value		0.328	0.749
*Age*			
≤30	1327 (42.3)	81.29 ± 22.39	8.15 ± 2.51
>30	1291 (57.7)	83.52 ± 21.59	7.96 ± 2.40
*P* value		0.010	0.049
*Highest education level*			
College degree or below	552 (21.2)	84.58 ± 22.56	7.91 ± 2.43
University degree or above	2055 (78.9)	81.80 ± 21.84	8.09 ± 2.47
*P* value		0.008	0.128
*Marital status*			
Single	1913 (73.1)	80.31 ± 22.85	8.32 ± 2.68
Married	705 (26.9)	83.15 ± 21.67	7.95 ± 2.37
*P* value		0.003	0.001
*Work years (years)*			
≤10	1534 (58.6)	81.27 ± 22.08	8.14 ± 2.47
>10	1084 (41.4)	83.97 ± 21.86	7.93 ± 2.44
*P* value		0.002	0.030
*Professional title*			
Nurse	1453 (55.5)	81.43 ± 22.39	8.12 ± 2.54
Nurse practitioner or above	1165 (44.5)	83.58 ± 21.51	7.97 ± 2.35
*P* value		0.013	0.120
*Annual salary ($)*			
≤8,430	1603 (61.2)	81.42 ± 22.54	7.90 ± 2.39
>8,430	1015 (38.8)	83.92 ± 21.12	8.29 ± 2.54
*P* value		0.005	<0.001

*Note*. Data are represented in *n* (percentage) or mean ± standard deviation.

**Table 2:** Mixed linear model analysis for predicting nurses' work engagement (*N* = 2,618).

Variables	Step 1: control variables		Step 2: explanatory variables	
	Beta (SE)	*P*	Beta (SE)	*P*
Age	0.20 (1.47)	0.889	1.06 (1.27)	0.404
Education level	−3.68 (1.11)	0.001	−2.01 (0.97)	0.038
Marital status	3.11 (1.14)	0.007	1.90 (0.99)	0.056
Work years	0.96 (1.48)	0.516	1.03 (1.28)	0.421
Professional title	1.02 (1.19)	0.390	1.02 (1.03)	0.324
Annual salary	1.38 (1.04)	0.185	0.69 (0.90)	0.442
Hospital hierarchy	0.00 (0.00)^a^	—	0.00 (0.00)^a^	—
Organizational support			0.57 (0.04)	<0.001
Leader empowerment			0.23 (0.05)	<0.001
Core self-evaluation			0.51 (0.06)	<0.001
∆Restricted log-likelihood			735.72	
Variance of hospital level	30.84 (13.88)	0.026	22.40 (10.07)	0.026
Residual	462.59 (12.85)	<0.001	347.72 (9.67)	<0.001

*Note*. Beta, unstandardized effect estimate. SE, standard error. ^a^This covariance parameter is redundant and the test statistic cannot be computed.

**Table 3:** Mixed linear model analysis for predicting patient-safety outcomes (*N* = 2,618).

Variables	Step 1: control variables		Step 2: explanatory variables		Step 3: interaction effect	
	Beta (SE)	*P*	Beta (SE)	*P*	Beta (SE)	*P*
Age	−0.03 (0.16)	0.875	−0.07 (0.15)	0.633	−0.06 (0.15)	0.677
Marital status	−0.35 (0.12)	0.005	−0.30 (0.12)	0.014	−0.30 (0.12)	0.012
Work years	−0.11 (0.16)	0.466	−0.11 (0.15)	0.477	−0.11 (0.15)	0.485
Annual salary	0.30 (0.11)	0.008	0.34 (0.11)	0.003	0.34 (0.11)	0.003
Hospital hierarchy	0.00 (0.00)^a^	—	0.01 (0.08)	0.904	0.01 (0.08)	0.945
Organizational support			−0.02 (0.01)	<0.001	−0.02 (0.01)	<0.001
Leader empowerment			−0.01 (0.01)	0.122	−0.07 (0.03)	0.012
Core self-evaluation			−0.03 (0.01)	<0.001	−0.10 (0.03)	0.001
Leader empowerment × core self-evaluation					0.001 (0.001)	0.029
∆Restricted log-likelihood			106.35		8.28	
Variance of hospital level	0.45 (0.19)	0.020	0.29 (0.16)	0.062	0.29 (0.16)	0.061
Residual	5.71 (0.16)	<0.001	5.45 (0.15)	<0.001	5.44 (0.15)	<0.001

*Note*. Beta, unstandardized effect estimate. SE, standard error. ^a^This covariance parameter is redundant and the test statistic cannot be computed.

## Data Availability

The data that support the findings of this study are available from the corresponding author upon reasonable request.
